# Biochemical Preparation of Cell Extract for Cell-Free Protein Synthesis without Physical Disruption

**DOI:** 10.1371/journal.pone.0154614

**Published:** 2016-04-29

**Authors:** Kei Fujiwara, Nobuhide Doi

**Affiliations:** Department of Biosciences & Informatics, Keio University, 3-14-1 Hiyoshi, Kohoku-ku, Yokohama 223–8522, Japan; Centro Nacional de Biotecnologia - CSIC / CIF Q2818002D, SPAIN

## Abstract

Cell-free protein synthesis (CFPS) is a powerful tool for the preparation of toxic proteins, directed protein evolution, and bottom-up synthetic biology. The transcription-translation machinery for CFPS is provided by cell extracts, which usually contain 20–30 mg/mL of proteins. In general, these cell extracts are prepared by physical disruption; however, this requires technical experience and special machinery. Here, we report a method to prepare cell extracts for CFPS using a biochemical method, which disrupts cells through the combination of lysozyme treatment, osmotic shock, and freeze-thaw cycles. The resulting cell extracts showed similar features to those obtained by physical disruption, and was able to synthesize active green fluorescent proteins in the presence of appropriate chemicals to a concentration of 20 μM (0.5 mg/mL).

## Introduction

Cell-free protein synthesis (CFPS) is an *in vitro* method to produce proteins from DNA using transcription-translation machinery derived from cells [1]. CFPS does not rely on living cells; therefore, it has been applied to various areas of research, to enable protein expression under artificial conditions. For example, it has been used successfully for *in vitro* preparation of membrane proteins [2–5], directed protein evolution[6–9], and construction of genetic circuits [10–12]. In addition, CFPS plays a very important role in bottom-up synthetic biology, a reconstructive approach to study biological systems [13–16]. Artificial cells are especially useful, as they entrap CFPS systems, and are used to mimic living cells and reveal physicochemical features [17–21]. Furthermore, CFPS has become an *in vitro* proteomics platform that does not rely on mass spectrometry [22–24]. Thus, this remarkable technology can enhance life science research.

Despite its versatility, the complexity of preparing CFPS systems has impeded its applications in research. CFPS requires transcription-translation machinery, DNA, and other small chemicals such as nutrients and energy sources [25]. Although recent efforts have made the preparation of each of these components easier [25–30], the initial setup remains challenging. The preparation of transcription-translation machinery from cells for *in vitro* use is an especially difficult technical hurdle.

The components of the transcription-translation machinery constitute approximately 30% of the CFPS mixture. Cell extracts (typically termed S30) are generally used to supply these components. Although a large amount of crude cell extract is needed, commercially available S30 is costly. Therefore, many researchers prepare this in the laboratory from cultured cells. The most commonly used tools for the preparation of cell lysates for CFPS are the French press and bead crushers [26,27,31,32]. However, this equipment is also expensive, and not generally available in the majority of laboratories. Recently, sonication-based methods for the preparation of cell extracts have been reported [25,28,30]. However, because sonication uses physical disruption, it has several unfavorable features such as temperature increase in samples during sonication, limitation of sample numbers, and increased preparation time with increasing sample volumes.

Biochemical disruption avoids the drawbacks described above. For example, lysozyme and osmotic treatment, followed by freeze-thaw cycles, is known to disrupt cells. After degradation of the cell wall by lysozymes, cells are easily disrupted by hypotonic treatment. Freeze-thaw cycles extract intracellular components by damaging the biological membranes. These methods have been widely used to prepare cell extracts to analyze recombinant protein expression; however, a recent report has shown that cell extracts prepared by lysozyme treatment or freeze-thawing do not show efficient CFPS activity [28].

Here, we report a method to prepare cell extract for CFPS using biochemical treatments. This method employs a combination of lysozyme treatment and freeze-thawing, with high-speed centrifuges being the only required machinery. The cell extracts prepared by our method were similar to those prepared by typical physical disruption methods and were capable of efficient CFPS. Because it is straightforward and scalable, our biochemical disruption method could provide an alternative to physical disruption methods developed so far.

## Materials and Methods

### Preparation of the LoFT (lysozyme treatment, osmotic shock, and freeze-thawing) cell extract

*Escherichia coli* BL21(DE3) codon plus (RIL) (Agilent) was used throughout the study. Overnight cell cultures in LB medium were inoculated in 1 L of fresh LB medium at concentrations ranging from 0.1% to 1% v/v. Cells were cultivated with shaking (120 rpm) at 37°C. After 1 h of culture, IPTG was supplied at a final concentration of 0.1 mM to induce expression of T7 RNA polymerase encoded in DE3 under the *lacUV5* promoter. Cells at OD_600_ = 1.0–2.0 were collected by centrifugation. The collected cells were suspended in 20 mL of 400 mM sucrose. At four time points, 50 μL of 20 mg/mL lysozyme (Nacalai Tesque, Inc., Kyoto, Japan), dissolved in 400 mM sucrose, was added to the cells suspensions (with a final lysozyme concentration of 0.2 mg/mL). Tubes were gently shaken by inversion after lysozyme addition and incubated on ice for 30 min. Next, the cells were washed twice with 20 mL of 400 mM pre-chilled sucrose. To prevent premature disruption during washing, cells were rapidly resuspended using a paintbrush rather than a pipette. The washed cells were collected by centrifugation, and dissolved in cold double distilled water (DDW). The amount of DDW varied as described in the manuscript text; however, typically 1 mL was used per g of wet cell paste. After DDW addition, the cells were rapidly transferred to 1.7 mL tubes and frozen in liquid nitrogen for 15 min or at -80°C for 1 h. Frozen cells were thawed in ice water (for approximately 1 h per mL of frozen solution). After thawing, and centrifugation at 25,000 × *g* for 1 h, the cell supernatants were collected as LoFT cell extracts and stored at -30°C.

The small molecules in the LoFT cell extract were exchanged with S30 buffer (5 mM Tris-HCl pH 7.6, 60 mM potassium glutamate, 14 mM magnesium acetate) using a filter unit. This process is sometimes unnecessary and was therefore only performed when needed. Briefly, 1 mL of the LoFT cell extract was transferred into an Amicon-Ultra 15 filter (10 kDa, MilliporeMerck). Next, 13 mL of the S30 buffer was added to the filter unit, which was centrifuged at 5,000 × *g* for 45 min (or longer) to reach a volume of less than 1.5 mL. This wash cycle was performed twice for the exchange of small molecules in the LoFT cell extract. This small molecule exchange process could be replaced with conventional dialysis using cellulose tubes.

### Plasmids

All DNA oligonucleotides used to construct the plasmids are listed in S1 Table.

The pOR2OR1-sfGFP-T500 plasmid was designed according to a previous report [27]. Briefly, through two rounds PCR, the OR2OR1 promoter (a strong0020σ70 promoter transcribed by bacterial RNA polymerase)[27], and a T7 g10 leader sequence (that enhances translation efficiency)[27] were ligated upstream of the gene encoding super folder green fluorescent protein (sfGFP) [33]. A T500 terminator was ligated downstream of the gene. In the first round of PCR, we used the primer set ORpFw1/ORpRv and pET15-sfGFP [25] as a template. In the second round of PCR, we used the amplified fragment as a template with ORpFw2/ORpRv primers. The PCR product was cloned into a BamHI-digested pUC19 plasmid by Gibson assembly (Gibson Assembly Master Mix, NEB, Ipswich, MA, USA).

To construct pET29-FtsZ, the *ftsZ* gene (a bacterial tubulin analog) was amplified by PCR using FtsZ-N/FtsZ-C as primers and pWARA2 [34] as a template. The PCR product was digested with NdeI and XhoI, and was cloned into the corresponding sites in pET29a (Merck Millipore, Darmstadt, Germany). To construct pET29-sfGFP, the NdeI/XhoI fragment of pET15-sfGFP was cloned into the NdeI/XhoI sites of pET29a. All plasmids were verified by DNA sequencing.

### CFPS reaction using LoFT cell extract

CFPS was carried out by mixing LoFT cell extract, template DNA, and reaction mixture (50 mM Hepes-KOH pH7.6, 36 mM 3-phosphoglyceric acids, 0.5 mM of each amino acid, 90 mM potassium glutamate, 14 mM magnesium acetate, 1.5 mM each of ATP and GTP, 0.9 mM each of CTP and UTP, 20 μg/ml *E*. *coli* tRNA mixture, 68 μM folinic acid, 0.75 mM cAMP, 0.33 mM NAD^+^, 0.26 mM CoA, 1 mM spermidine, 12 mM maltose, 2% PEG8000, and 1 mM IPTG), in a total volume of 5 μL (except for omission assays wherein a total volume of 10 μL was used). Plasmid concentrations were 1.5 nM for genes using the T7 promoter and 10 nM for genes under control of the OR2OR1 promoter. The final concentration of the LoFT cell extract was 10 mg/mL. Linear DNA was prepared by PCR using the following primer sets: ORpFw2/ORpRv for pOR2OR1-sfGFP, and T7pUp/T7tDOWN (S1 Table) for pET29-sfGFP. DNA used in this study was purified using the Mini Plus™ Plasmid DNA Extraction System (VIOGENE, New Taipei City, Taiwan) or QIAprep Spin Miniprep Kit (QIAGEN, Venlo, Netherlands). All CFPS reactions were performed at 29°C. The concentration of sfGFP synthesized was estimated by detecting total florescence levels (Safire microplate reader, TECAN, Männedorf, Switzerland), using histidine-tagged sfGFP purified by Ni-NTA agarose (QIAGEN) and a gel filtration column (HiPrep 16/60 Sephacryl S-200 HR, GE healthcare, IL, USA) as a standard.

## Results

### Crude cell extracts prepared by lysozyme treatment and freeze-thawing (LoFT cell extract)

**Fig 1:**
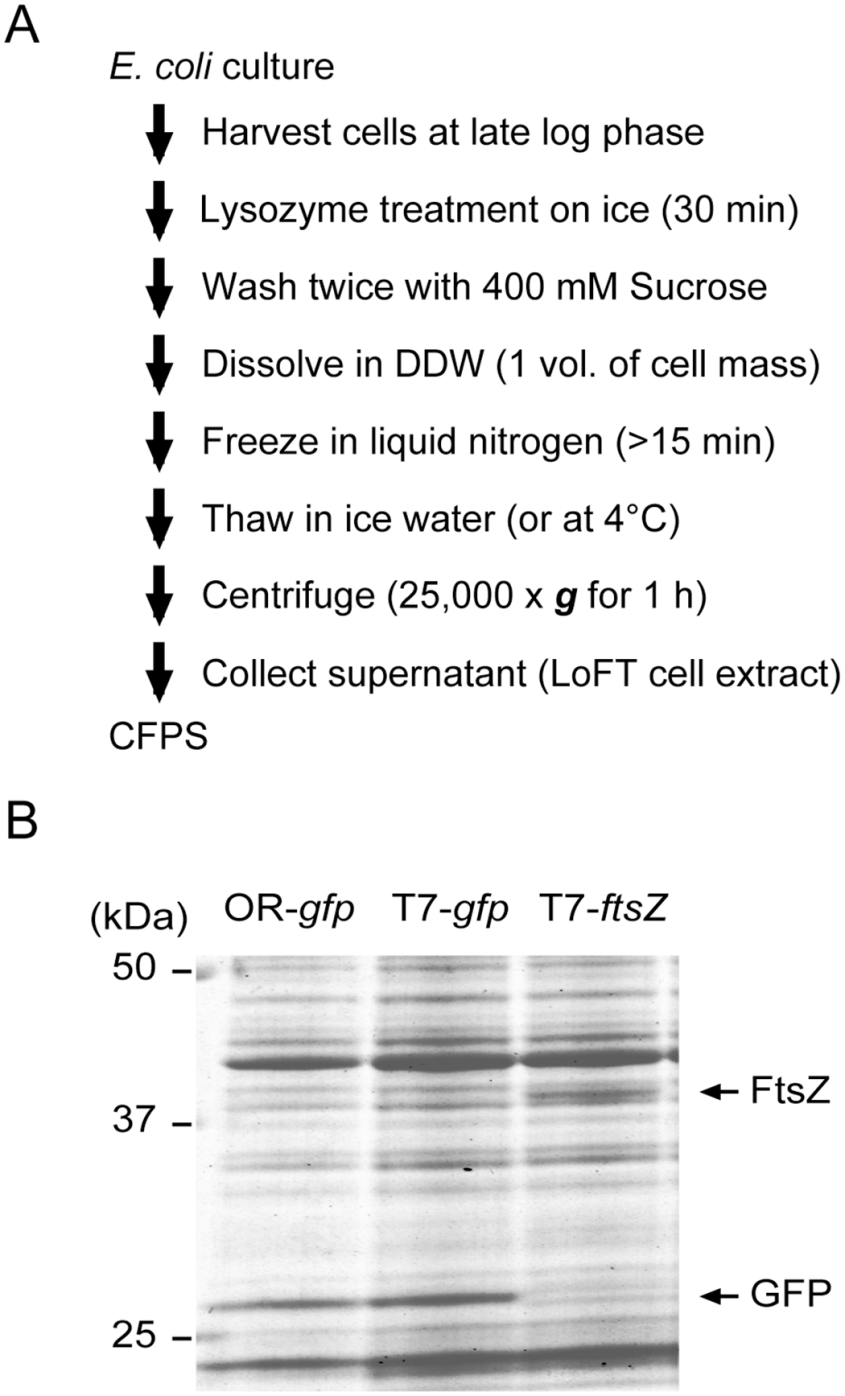
LoFT (lysozyme treatment, osmotic shock, and freeze-thawing) cell extraction protocol. (A) A typical protocol to prepare the LoFT cell extract. (B) Coomassie staining after SDS-PAGE, showing the expression of GFP and FtsZ (a bacterial tubulin homolog) using the LoFT cell extraction protocol. pOR2OR1-sfGFP (*gfp* under control of the OR2-OR1 promoter), pET29-sfGFP (*gfp* under control of the T7 promoter), or pET29-ftsZ (*ftsZ* under control of the T7 promoter) plasmid was mixed with LoFT cell extract and reaction mixtures and incubated for 14 h at 29°C. Expression of FtsZ was confirmed using a FluorTect GreenLys *in vitro* translation labeling system (Promega, Fitchburg, WI).

*E*. *coli* cells treated with lysozyme are fragile and easily disrupted by osmotic shock or freeze-thaw cycles. Thus, we combined these procedures to prepare highly concentrated cell extracts, as described in Fig 1A. One L culture of *E*. *coli* cells at late log-phase (OD_600_ = 1.0–2.0) was treated with lysozyme on ice. Cell were then washed with 400 mM sucrose and resuspended in DDW. The cells were frozen in liquid nitrogen and were gradually thawed in ice water, and supernatants of the treated cells were collected by centrifugation. Because the pellets were very viscous, the supernatants were carefully collected. The resultant cell extracts, obtained by lysozyme treatment, osmotic shock, and freeze-thawing (LoFT cell extract), typically contained 20–30 mg/mL of proteins.

LoFT cell extracts exhibited efficient protein expression in the CFPS system, when combined with other reaction mixture components and plasmid DNA (Fig 1B). Typically, approximately 10–20 μM (0.25–0.5 mg/mL) of active sfGFP was synthesized after a 14 h reaction at 29°C. Both T7 RNA polymerase and endogenous *E*. *coli* RNA polymerase in the LoFT cell extract worked as transcription machinery for CFPS (Fig 1B). The FtsZ protein, a bacterial tubulin analog, was also synthesized by CFPS using LoFT cell extract (Fig 1B, S1 Fig).

Protein expression levels were nearly saturated after a 3 h reaction (S2 Fig). Both 3-PGA and creatine phosphate kinase were able to be used as energy sources (S3 Fig) as reported in other CFPS studies [25–28,30,31].

### Optimal conditions of freeze-thaw after lysozyme treatment

**Fig 2:**
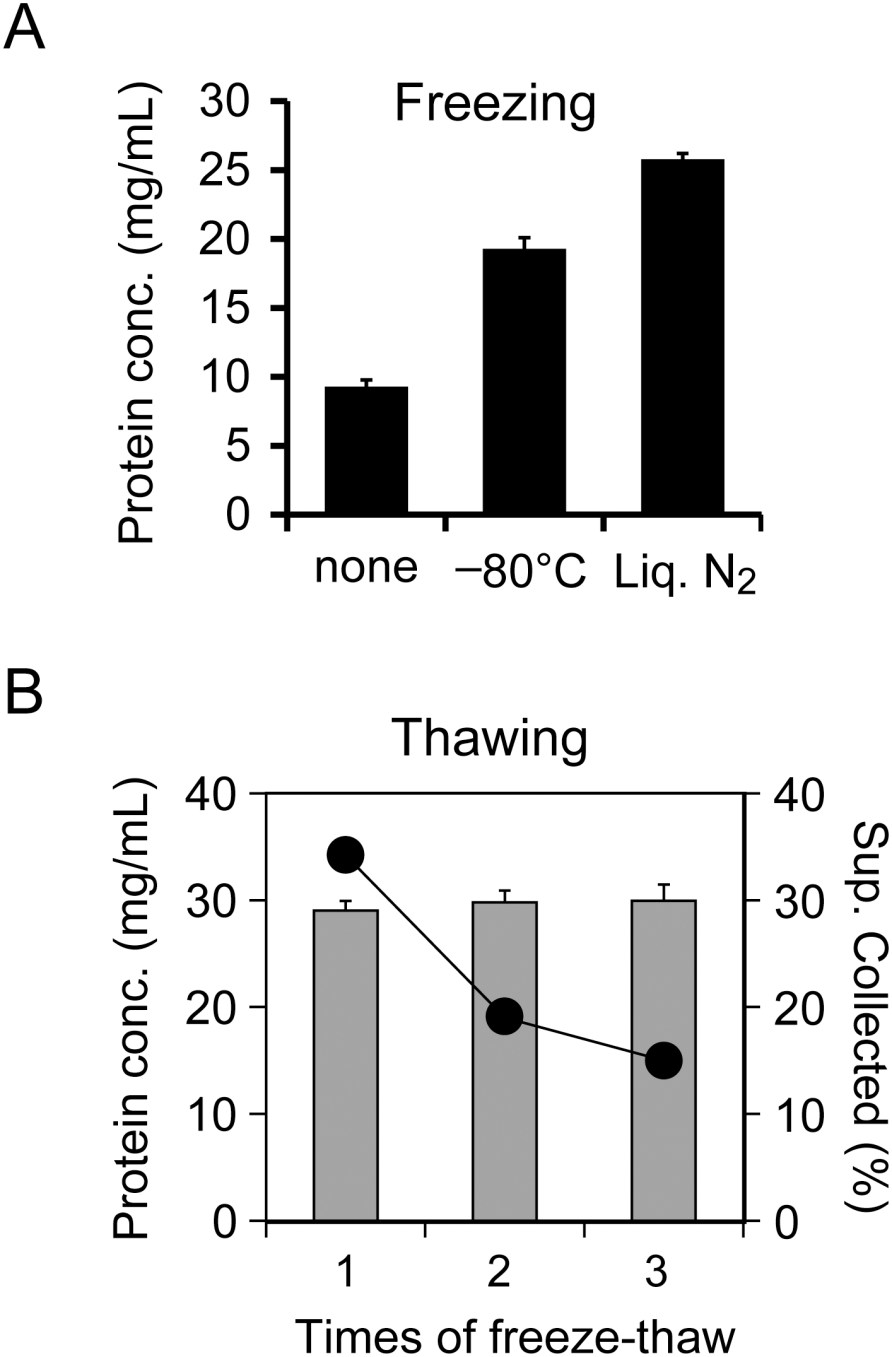
Conditions of freezing and thawing for LoFT cell extraction. (A) Effects of freezing temperature on protein concentration (protein conc.) after freeze-thawing. Samples were frozen in a deep freezer (-80°C) for 1 h, immersed in liquid nitrogen (Liq. N_2_) for 15 min, or not frozen. (B) Protein concentration (gray bars) in the LoFT cell extracts and volume of supernatant (Sup.) after centrifugation (circles) compared to the number of freeze-thaw cycles. Collectable supernatant volumes were derived from the viscosity of cell debris after freeze-thaw. Because the standard deviations of the collectable solution (n = 3) were smaller (on the scale shown) than the circle sizes, error bars are not indicated. All samples in (A) and (B) were thawed by incubation in ice water, and error bars indicate standard deviation (n = 3).

The freezing conditions and the number of cycles are important factors to maximize protein yield during freeze-thaw disruption. First, we evaluated the effect of freezing temperatures on the protein yield. Cell suspensions were divided into six tubes immediately before freezing; three tubes were frozen in a deep freezer (at -80°C) for 1 h, whereas the other three tubes were immersed in liquid nitrogen (at -196°C) for 15 min. All tubes were thawed in ice water, and supernatants (cell extracts) were collected after centrifugation. In terms of protein concentration, we found that freezing in liquid nitrogen, compared to freezing at -80°C, resulted in a 1.33-fold higher protein yield (Fig 2A).

Second, the effect of multiple freeze-thaw cycles on protein yield was evaluated. Specifically, one, two, and three freeze-thaw cycles were tested. The number of cycles did not affect the protein concentrations of the LoFT cell extracts. However, multiple freeze-thaw cycles did reduce the volume of collectable supernatant after centrifugation (Fig 2B). Therefore, these results indicated that a single freeze-thaw step was optimal for the preparation of LoFT cell extracts when using DDW as the solution for osmotic shock.

### Effect of double distilled water to wet cell ratio on protein yields

Next, we examined the relationship between protein yields and the DDW volume added before freezing. LoFT cell extracts were prepared using 0.5, 1.0, 1.5, 2.0, and 5.0 mL of DDW per 1 g of wet cells, and their respective protein concentrations and total protein yields were evaluated.

Smaller volumes of DDW led to higher protein concentrations in the LoFT cell extracts (S4A Fig). The total protein yields were not equal among the different conditions, and the highest yield was obtained using 1.5 mL of DDW (S4B Fig); however, protein concentration did not reach the concentration required for CFPS (>20 mg/mL). These results suggest that 1.0 mL DDW per 1 g cells is the recommended ratio, because this volume gives sufficient protein concentrations for CFPS while protein yields remain relatively high.

### Effect of relative centrifugal force on the LoFT cell extract

**Fig 3:**
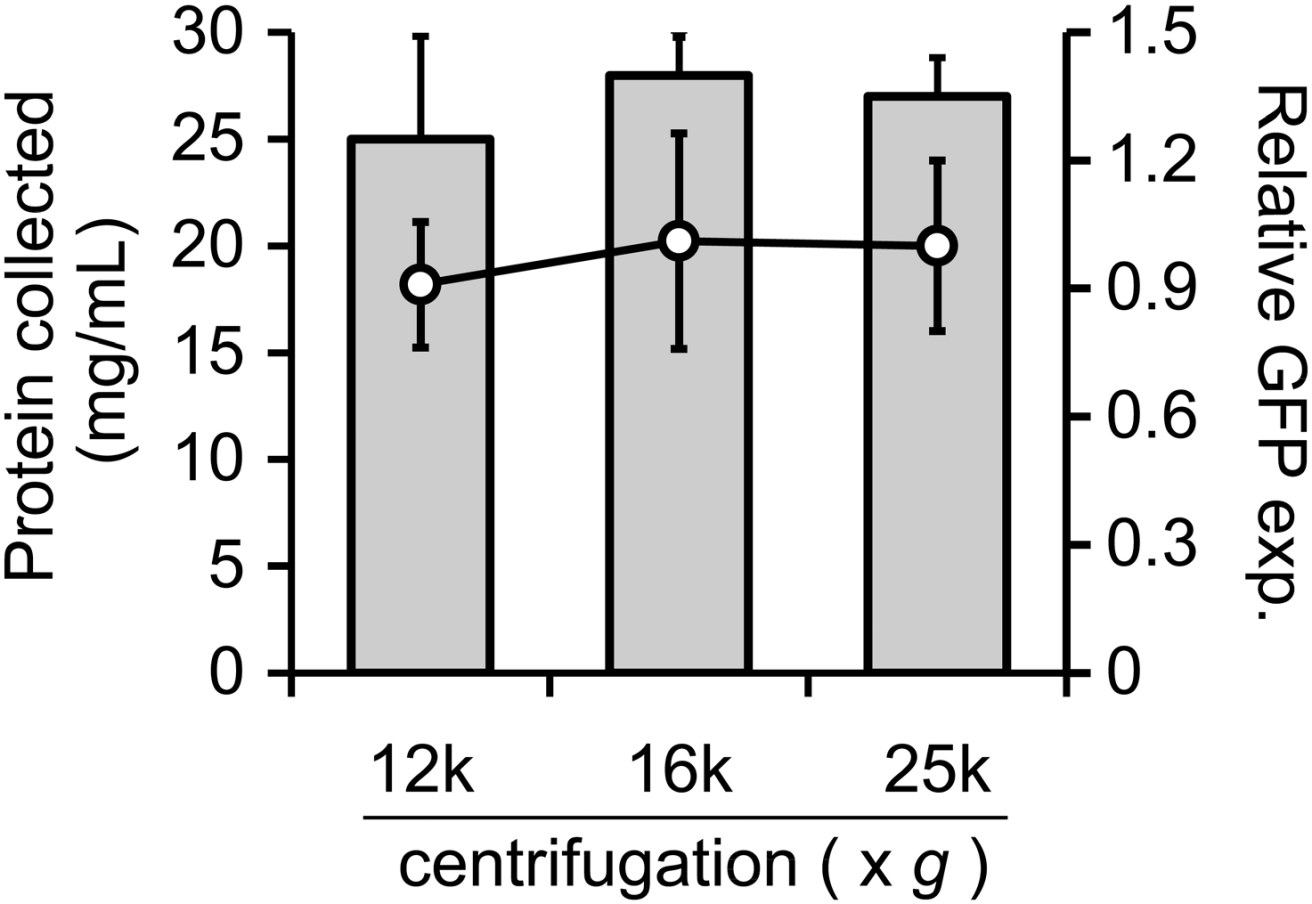
Effect of centrifugation force on LoFT cell extract. Protein concentration (gray bars) and levels of sfGFP expression, from CFPS using LoFT cell extracts and 3 nM pOR2OR1-sfGFP (circles), were plotted against forces of centrifugation (12,000 × *g* for 2.5 h, 16,000 × *g* for 94 min, and 25,000 × *g* for 1 h) after freeze-thawing. Error bars indicate standard deviations (n = 3). Expression levels of sfGFP were normalized to the average value of the cell extracts obtained after centrifugation at 25,000 × *g* for 1 h (vertical axis). GFP exp. means “GFP expression”.

Relative centrifugal force (rcf) is an important factor for the preparation of cell extracts for CFPS. Usually, supernatants are obtained after centrifugation at 30,000 × *g* for 30–60 min (S30); our LoFT extracts were prepared using centrifugation at 25,000 × *g* for 60 min. However, commonly used centrifuges for 1.7 mL tubes typically do not generate more than 20,000 × *g*. To date, several groups have reported that centrifugation at 12,000 × *g* is sufficient to collect cell extracts after using physical disruption methods [26,30]. Therefore, we evaluated the effect of rcf on LoFT cell extract preparation. Centrifugation levels were set to 12,000 × *g* and 16,000 × *g*, speeds that can be generated in typical microcentrifuges. The centrifugation time was adjusted to achieve equal levels of total centrifugation force (rcf × time), compared to 25,000 × *g* for 60 min. Our results demonstrated that the extracted protein yields and concentrations were similar for all tested rcf conditions (Fig 3). CFPS using the LoFT cell extracts prepared at lower centrifugation speeds exhibited activity levels comparable to those observed for extracts obtained by centrifugation at 25,000 × *g* for 60 min.

### Essential small molecules for CFPS using the LoFT cell extract

**Fig 4:**
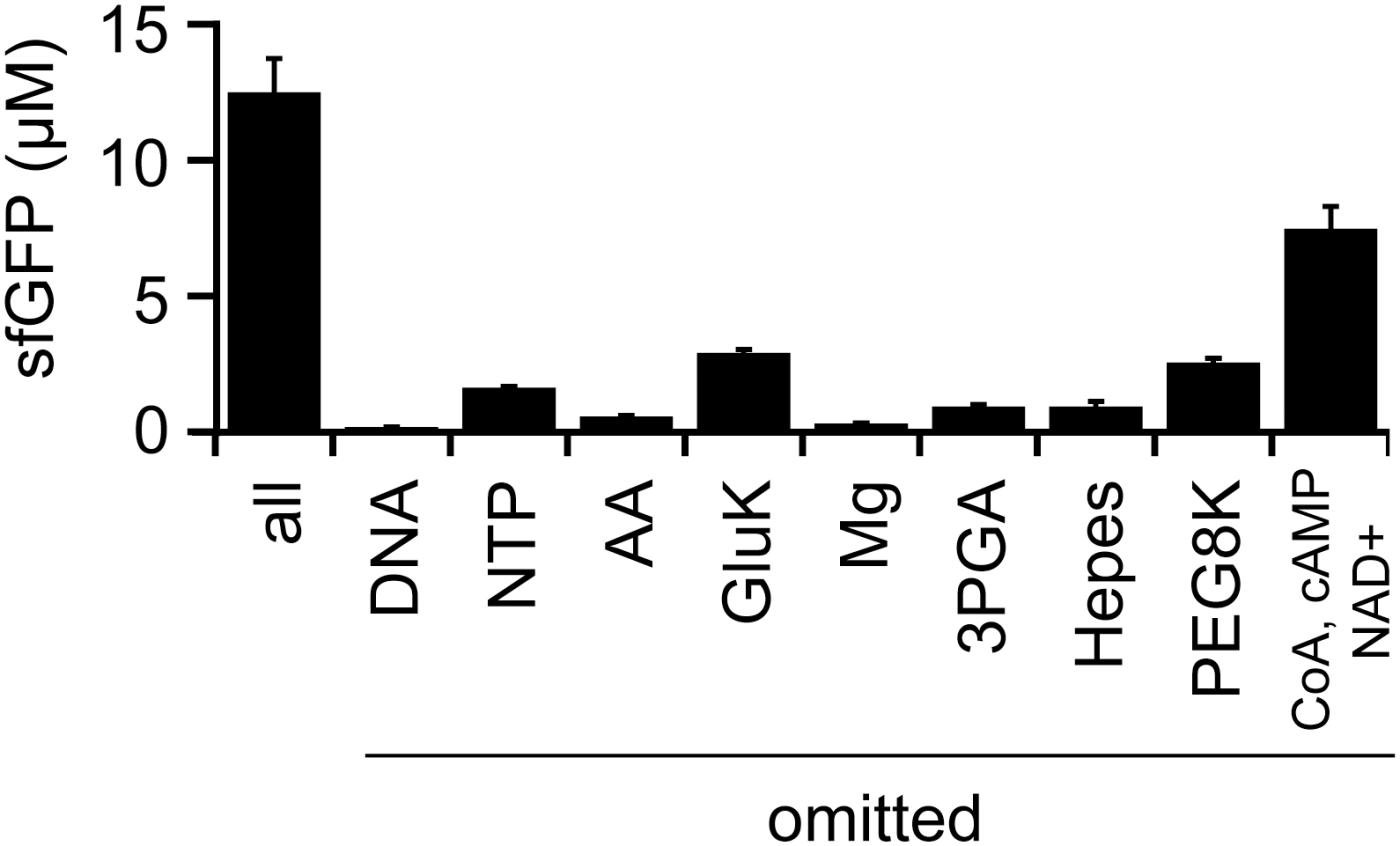
Essential chemicals for CFPS using the LoFT cell extract. The importance of each chemical for GFP expression was evaluated by systematic omission from the reaction mixtures. AA indicates the mixture of 20 amino acids. Template DNA used was 10 nM pOR2OR1-sfGFP. CFPS was performed at 29°C for 14 h. In the condition indicated by “all,” none of the chemicals were omitted. Error bars indicate standard deviations (n = 4).

The methods to prepare LoFT cell extract do not include the removal of small molecules. Our previous studies on additive-free cell extracts prepared by sonication indicated that nucleoside triphosphates (NTPs) and amino acids can be omitted from the CFPS reaction mixture [25]. Thus, the importance of each component in the CFPS reaction mixture was evaluated in an omission assay (Fig 4). The components in our reaction mixture were template DNA (GFP gene under the OR2-OR1 promoter [27]), NTPs, amino acids, potassium glutamate (GluK), magnesium acetate (Mg), 3-phospho glycerate (3PGA), HEPES buffer, PEG8000, maltose, CoA, cAMP, NAD^+^, tRNA, spermidine, and formyl donor. Omission of cAMP, NAD^+^, and CoA affected the efficiency of CFPS, as previously reported elsewhere [35]. The importance of tRNA, spermidine, formyl donor, and maltose for CFPS varied among the different LoFT cell extracts; thus, we cannot conclusively determine the importance of these chemicals. Other chemicals were determined to be indispensable for efficient CFPS based on the omission assay (Fig 4). These results suggest that LoFT cell extract requires more species of small metabolites for CFPS than additive-free cell extract prepared by sonication [25]. This might have resulted from leakage of small molecules during the washing steps after lysozyme treatment.

### CFPS from linear DNA fragments using the LoFT cell extract

**Fig 5:**
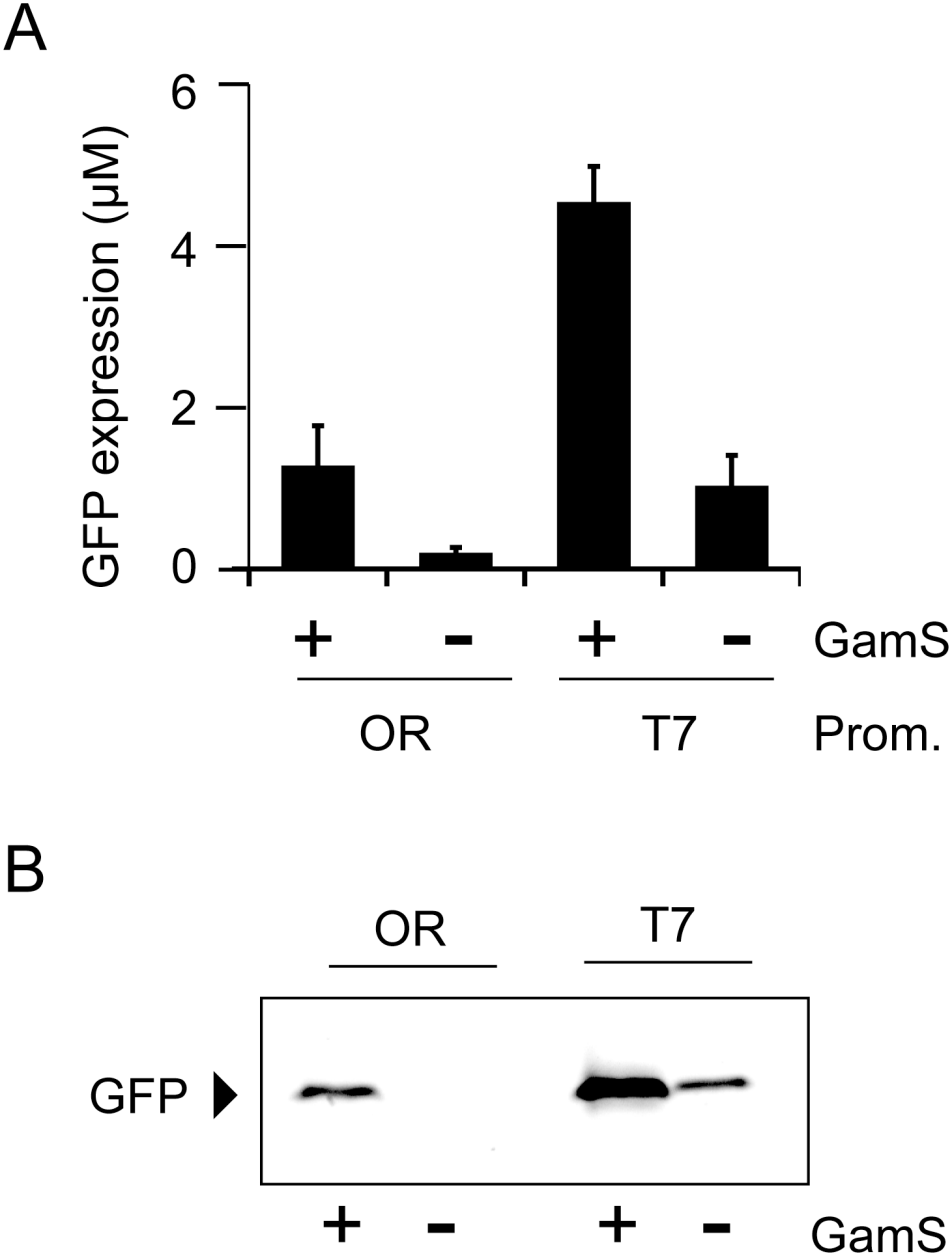
CFPS using a linear DNA template and LoFT cell extract. sfGFP was produced using LoFT cell extract and 5 nM linear DNA (PCR product) for 1 h at 29°C, in the absence (−) or presence (+) of 4 μM GamS. OR and T7 indicate PCR products from the OR2OR1-sfGFP (from the pOR2OR1-sfGFP plasmid) and T7-sfGFP (from the pET29-sfGFP plasmid), respectively. Prom. means “Promoter”. (A) Expression levels of GFP estimated by total fluorescence levels. Error bars indicate standard deviations (n = 3). (B) GFP fluorescence detected using SDS-PAGE without boiling. Only bands corresponding to GFP fluorescence are shown.

For applications in bottom-up synthetic biology, CFPS from linear DNA produced by PCR should be possible [12]. Previous reports have shown that the addition of GamS, an inhibitor of RecBCD nuclease, enables the use of linear DNA as a template for this process [36]. We confirmed that the GamS-linear DNA system also works with LoFT cell extract. Fluorescence intensity measurement showed that LoFT cell extract is able to produce GFP, encoded by a linear DNA fragment, in a GamS-dependent manner; however, the CFPS activity was several times lower when compared to CFPS performed using circular plasmids (Fig 5A). In addition, protein expression stopped within 1 h (S5 Fig). GamS supplementation slightly, but not effectively, reduced GFP expression in CFPS using circular plasmids (S6 Fig). The lower CFPS activity using linear DNA with the GamS system was also reported in a previous study using S30 prepared by conventional physical disruption [12]. GFP fluorescence after SDS-PAGE without boiling supported these results (Fig 5B).

### Small molecules in the LoFT cell extract could be exchanged with buffer

**Fig 6:**
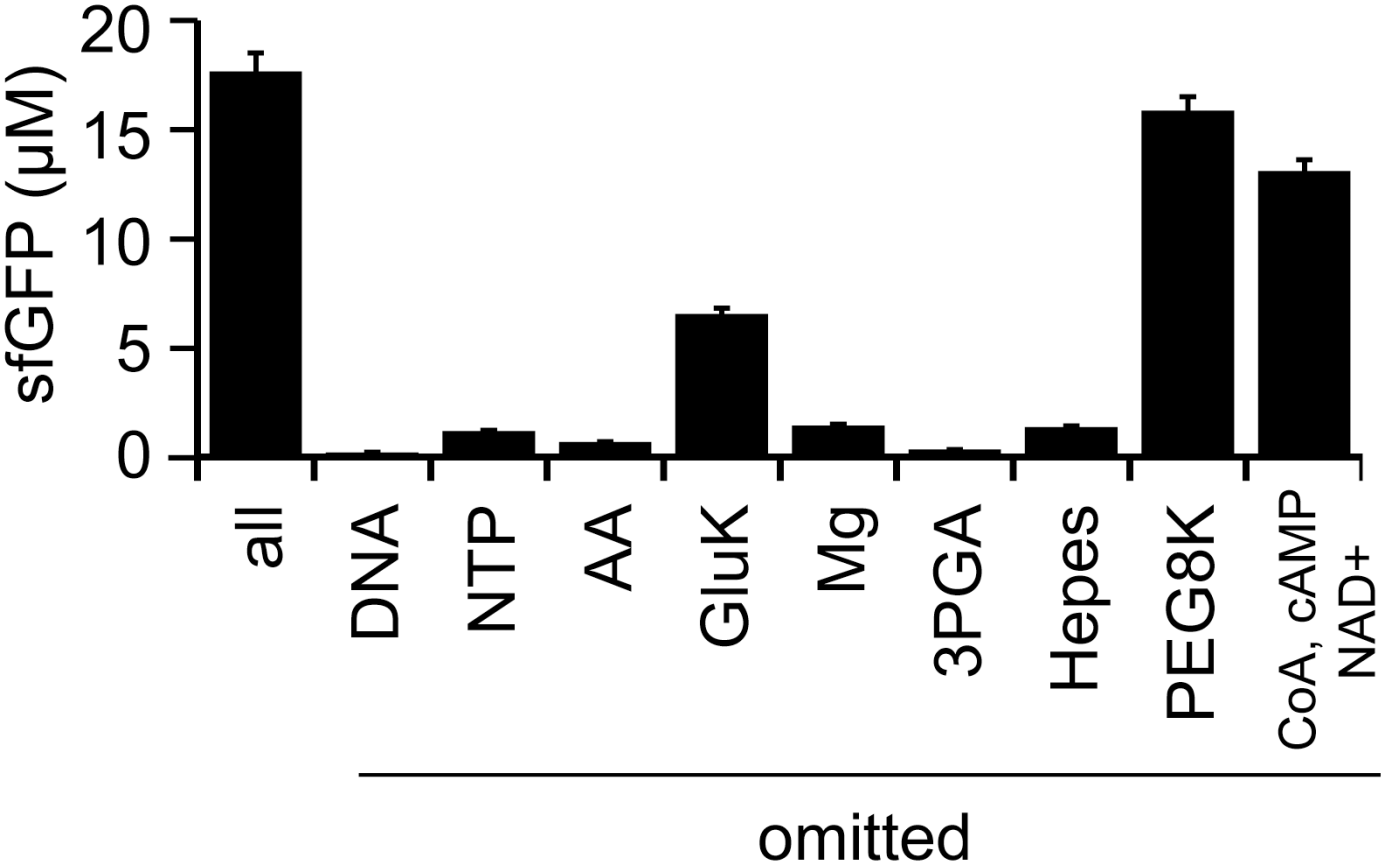
Essential chemicals for CFPS using LoFT cell extract after buffer exchange. The importance of each chemical for GFP expression was evaluated by systematic omission from the reaction mixtures. AA indicates the mixture of 20 amino acids. pOR2OR1-sfGFP (10 nM) was used as the DNA template. CFPS was performed at 29°C for 14 h. “all” indicates no chemicals were omitted. Expression levels of sfGFP were normalized to the average value of “all.” Error bars indicate standard deviation (n = 4). Expression of sfGFP without GluK and Mg is a result of the exchange buffer (S30 buffer) containing these chemicals.

The procedure to prepare LoFT cell extract does not require any buffers. However, the use of a buffer could perhaps improve the efficiency of CFPS. To address this point, we tested if buffer exchange, after preparation of LoFT cell extract, could improve CFPS efficiency. After small molecules in the LoFT cell extract were exchanged with CFPS buffer (5 mM Tris-HCl pH 7.6, 60 mM potassium glutamate, 14 mM magnesium acetate), the activity was assayed using GFP as a reporter. The omission assay revealed that the dependence on added chemicals, for CFPS activity, was quite similar to that of extracts without buffer exchange, with the exception of PEG8000 (Fig 6).

We also tested the effect of LoFT cell extraction using buffers, instead of DDW, on CFPS. DDW at the last suspension step was replaced with S30 buffer (see Materials & Methods). Because several hundred microliters of cell suspension was used, this replacement slightly decreased protein concentrations after centrifugation. In addition, the supernatant obtained after a single freeze-thaw cycle showed reduced CFPS activity (S7 Fig). Interestingly, CFPS activity was recovered in supernatants that underwent two freeze-thaw cycles (S7 Fig). However, we also found that a single freeze-thaw cycle was sufficient when CFPS buffer was used at a volume of 1.5 times the cell mass.

## Discussion

In this study, we report a cell extract preparation method for CFPS based on biochemical treatment (LoFT cell extract). Expensive equipment for physical disruption, which is typically used to prepare cell extracts for CFPS, is not required using this method. The only essential materials for the preparation of LoFT cell extract are liquid nitrogen, lysozymes, and centrifuges. These materials are relatively cheap ($0.15 to prepare cell extract from 1 L culture) and commonly available in laboratories.

A comparison of cell-free extracts generated by physical disruption and by the LoFT method is summarized in Table 1. The maximum protein yield after CFPS using LoFT cell extract is lower compared to other methods, and the time to prepare cell extract is average among the methods tested. Instead, the LoFT method is effective for small-scale culture, less than 1 L, similar to sonication methods [25,28,30]. This is in contrast to other methods that require more than 3 L of culture [26,29,31]. Since the LoFT method does not require physical disruption, it is not necessary to control the temperature of the sample or the power of the equipment, as is required using other methods of CFPS preparation to avoid denaturation of proteins. Furthermore, the LoFT preparation can be performed for multiple samples in parallel, which is difficult for procedures utilizing equipment for physical disruption.

**Table 1:** A comparison of cell-free extracts prepared by physical disruption and by the LoFT method.

	Physical disruption						Biochemical preparation
Disruption methods	Bead	Bead Mill	French press	Soncation	Soncation	Soncation	LoFT
Reference	F Caschera et al. (2014) Biochimie^[^^29^^]^	T Kigawa et al., (2004) J Struct Funct Genomics^[^^31^^]^	TW Kim et al., (2006) J Biotechnol^[^^26^^]^	P Shrestha et al., (2012) Biotechniques^[^^28^^]^	K Fujiwara et al.,(2013) PLOS ONE^[^^25^^]^	YC Kwon et al., (2015) Sci Rep^[^^30^^]^	This study
Maximum protein yield (mg/mL)	1.5	0.8	0.7	1.0	0.1	0.5	0.5
Preparation time after cell collection	8h	6h	2h	1.5h	3h	20min	3h
Culture scale less than 1L	No	No	No	Yes	Yes	Yes	Yes
Physical disruption machine	required	required	required	required	reqired	required	not required
Thermal and power control	critical	critical	critical	critical	critical	critical	not required
Pararell preparation	No	No	No	No	No	No	Yes

In spite of the versatile applications of CFPS, the initial cost has been a barrier for its widespread use. The LoFT method effectively reduces this limitation. However, one remaining challenge is the mixing of many small molecules for the CFPS reaction. Recent efforts by other groups have led to more straightforward and cost-effective methods to prepare amino acids and energy producing systems for CFPS [37–40]. Continuing to refine these methods for the preparation of reaction mixtures will pave the way for the widespread use of CFPS in biomedical research.

The preparation method for CFPS established in the present study resembles the method used to prepare cell extracts for genomic DNA replication studies [41]. Both methods use lysozymes, liquid nitrogen, and centrifuges. The protocol to prepare the cell extract for genomic DNA replication was designed to isolate intracellular components from hundreds of liters of cell culture. Although 9 L of cell culture was the largest volume that we tested, we expect that the LoFT cell extract preparation method will be scalable to hundreds of liters of *E*. *coli* culture for industrial applications.

## Supporting Information

S1 Fig Detection of newly synthesized FtsZ using an *in vitro* labeling kit. Expression of FtsZ after the CFPS reaction was observed using FluorTect GreenLys *in vitro* translation labeling system (Promega). For this experiment, tRNA aminoacylated with fluorescence-labeled lysine was added to the CFPS mixtures in accordance with the manufacturer’s instructions. Total and Sup. indicate whole and supernatant fraction, respectively, of the CFPS reaction mixture after centrifugation at 20000 × *g* for 30 min.
(TIF)

S2 Fig Time course of GFP expression using the LoFT extract. Relative GFP expression levels during the CFPS reaction were plotted. CFPS was performed at 29°C for 1, 3, 6, and 14 h. Error bars indicate standard deviation (n = 4). Triangles (dashed line) and filled circles (solid line) indicate the CFPS reaction using OR-*gfp* (pOR2OR1-sfGFP) or T7-*gfp* (pET29-sfGFP) as a template, respectively. Expression levels of sfGFP were normalized to the average value of sfGFP levels after the 14 h reaction using pOR2OR1-sfGFP-T500.
(TIF)

S3 Fig Creatine phosphate and kinase system works as an energy source of CFPS when using LoFT cell extract. CP-CK indicates the energy recycling system using creatine phosphate and creatine kinase. In the case of CP-CK, cAMP, CoA, NAD^+^, 3-PGA and maltose were omitted from the reaction mixture in accordance with the method reported in a previous study [25]. Specifically, the CFPS reaction mixture contained 50 mM Hepes-KOH pH7.6, 40 mM creatine phosphate, 0.5 mM of each amino acid, 90 mM potassium glutamate, 14 mM magnesium acetate, 1.5 mM each of ATP and GTP, 0.9 mM each of CTP and UTP, 20 μg/ml *E*. *coli* tRNA mixture, 68 μM folinic acid, 1 mM spermidine, 2% PEG8000, 1 mM IPTG, and 100 μg/ml creatine kinase. Creatine kinase from rabbit muscle was purchased from Oriental Yeast Co., Ltd (Tokyo, Japan).
(TIF)

S4 Fig Relationship between protein yields and DDW volume added before freezing. Protein concentration (A) and total protein yield (B) after extraction using 0.5, 1.0, 1.5, 2.0, and 5.0 mL of DDW per 1 g of wet cells are shown. The value obtained for 1.0 mL DDW per 1 g wet cells was set to 1.0.
(TIF)

S5 Fig Protein expression stops at an early time point when using linear DNA and the GamS system. Relative GFP expression levels during the CFPS reaction were plotted. CFPS was performed at 29°C for 1 h or 3 h. Expression levels of sfGFP were normalized to the average value of the “1 h” condition. Error bars indicate standard deviation (n = 4). For template DNA, 5 nM of PCR product of pOR2OR1-sfGFP-T500 was used.
(TIF)

S6 Fig GamS does not enhance protein expression when using plasmid DNA. Relative sfGFP expression levels after the CFPS reaction 29°C for 14 h with or without GamS are shown. Expression levels of sfGFP were normalized to the average value of “GamS-.” Error bars indicate standard deviation (n = 4).
(TIF)

S7 Fig Productivity of LoFT cell extract using buffers instead of DDW. Productivity was assessed by measuring the levels of sfGFP expression after CFPS using LoFT cell extracts and 10 nM pOR2OR1-sfGFP. Error bars indicate standard deviation (n = 3). Expression of sfGFP, in the LoFT cell extract using DDW, was set as 1.
(TIF)

S1 Table DNA primers used in this study. (DOCX)
