# Supplementary figures and images for: Biochemical Preparation of Cell Extract for Cell-Free Protein Synthesis without Physical Disruption
Source: PLoS One. 2016 Apr 29;11(4):e0154614. doi: 10.1371/journal.pone.0154614 (PMC4851396; doi:10.1371/journal.pone.0154614)

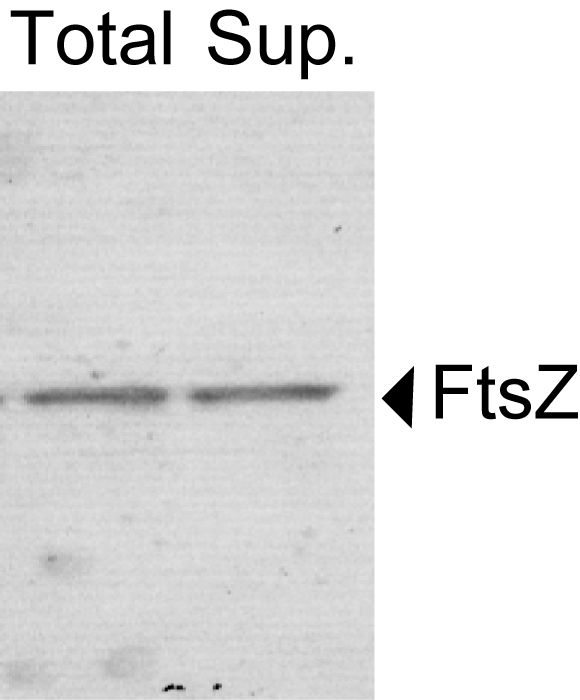

Supplement: S1 Fig — Expression of FtsZ after the CFPS reaction was observed using FluorTect GreenLys in vitro translation labeling system (Promega). For this experiment, tRNA aminoacylated with fluorescence-labeled lysine was added to the CFPS mixtures in accordance with the manufacturer’s instructions. Total and Sup. indicate whole and supernatant fraction, respectively, of the CFPS reaction mixture after centrifugation at 20000 × g for 30 min. (TIF) [file pone.0154614.s001.tif]

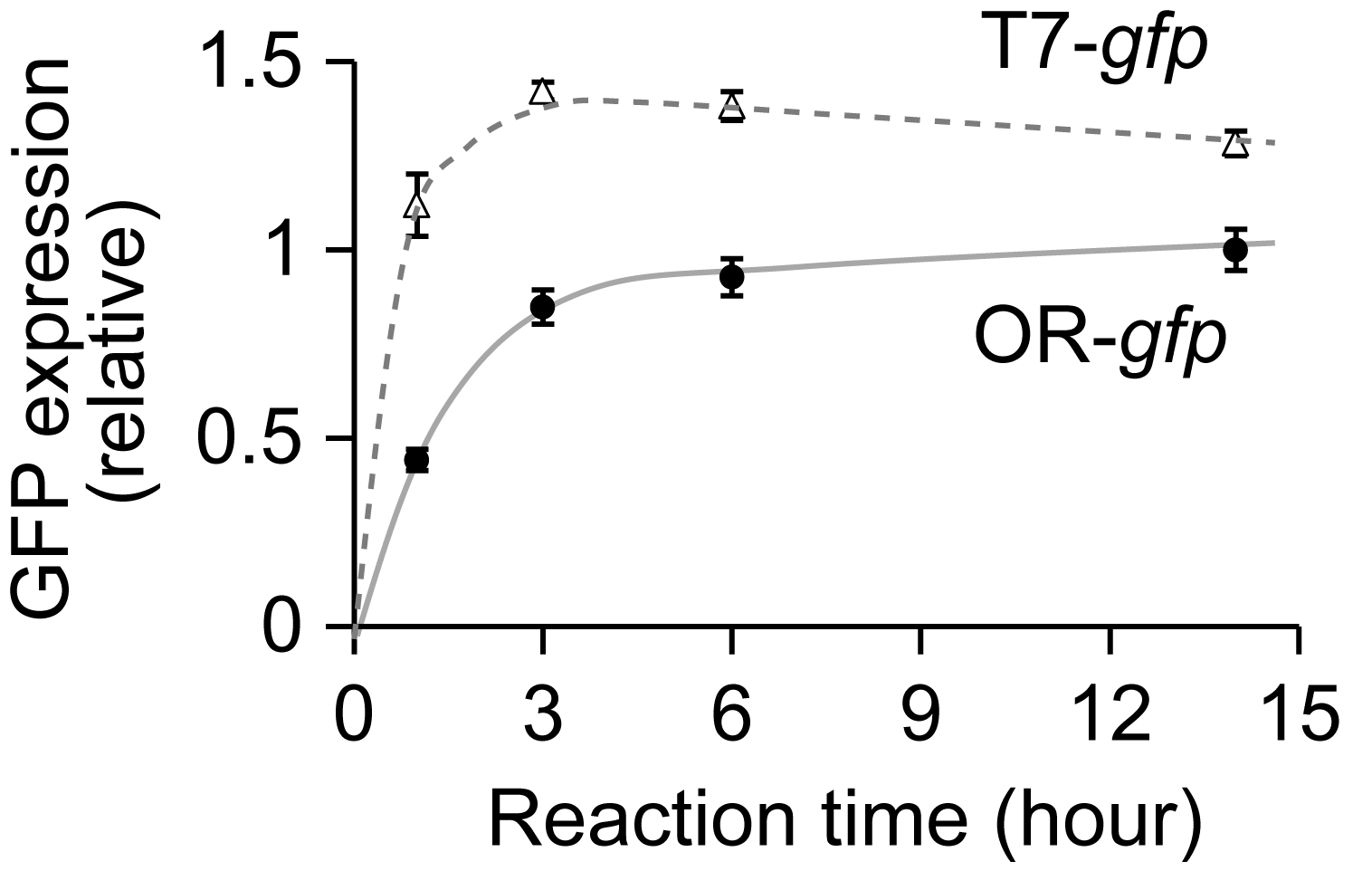

Supplement: S2 Fig — Relative GFP expression levels during the CFPS reaction were plotted. CFPS was performed at 29°C for 1, 3, 6, and 14 h. Error bars indicate standard deviation (n = 4). Triangles (dashed line) and filled circles (solid line) indicate the CFPS reaction using OR-gfp (pOR2OR1-sfGFP) or T7-gfp (pET29-sfGFP) as a template, respectively. Expression levels of sfGFP were normalized to the average value of sfGFP levels after the 14 h reaction using pOR2OR1-sfGFP-T500. (TIF) [file pone.0154614.s002.tif]

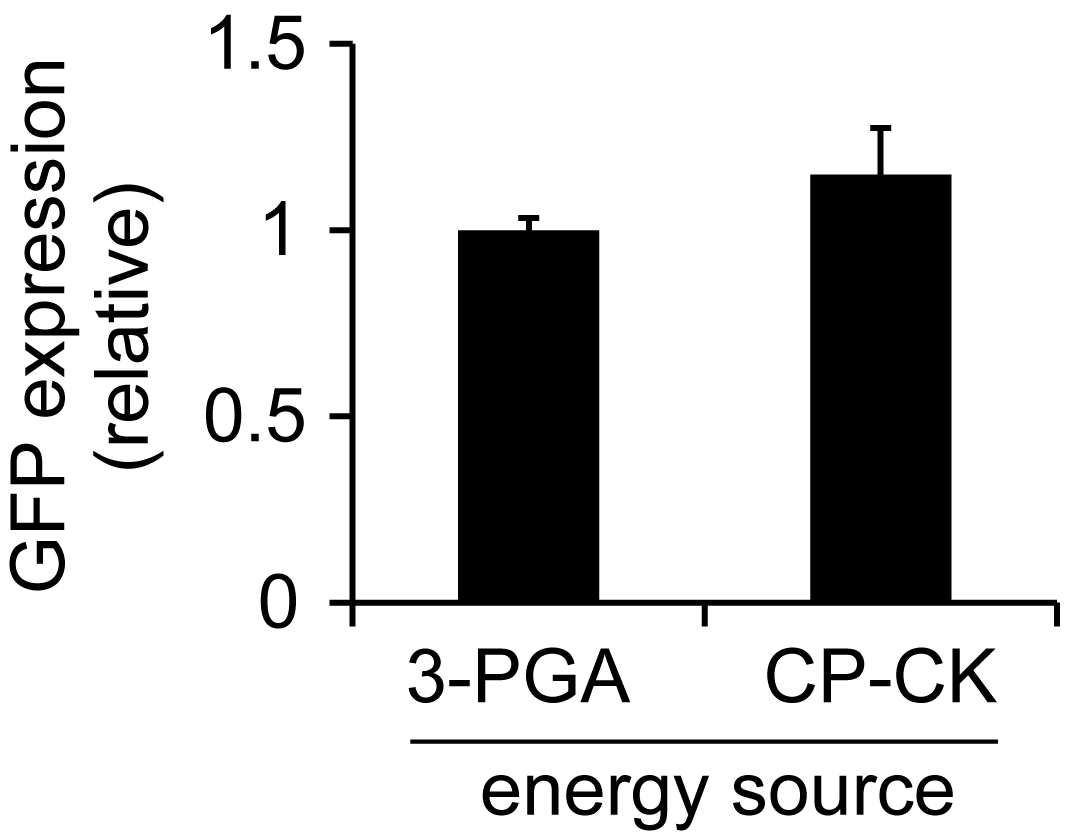

Supplement: S3 Fig — CP-CK indicates the energy recycling system using creatine phosphate and creatine kinase. In the case of CP-CK, cAMP, CoA, NAD+, 3-PGA and maltose were omitted from the reaction mixture in accordance with the method reported in a previous study [25]. Specifically, the CFPS reaction mixture contained 50 mM Hepes-KOH pH7.6, 40 mM creatine phosphate, 0.5 mM of each amino acid, 90 mM potassium glutamate, 14 mM magnesium acetate, 1.5 mM each of ATP and GTP, 0.9 mM each of CTP and UTP, 20 μg/ml E. coli tRNA mixture, 68 μM folinic acid, 1 mM spermidine, 2% PEG8000, 1 mM IPTG, and 100 μg/ml creatine kinase. Creatine kinase from rabbit muscle was purchased from Oriental Yeast Co., Ltd (Tokyo, Japan). (TIF) [file pone.0154614.s003.tif]

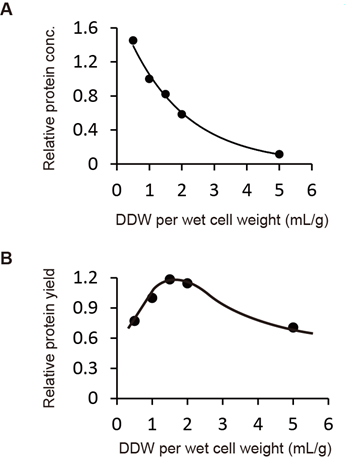

Supplement: S4 Fig — Protein concentration (A) and total protein yield (B) after extraction using 0.5, 1.0, 1.5, 2.0, and 5.0 mL of DDW per 1 g of wet cells are shown. The value obtained for 1.0 mL DDW per 1 g wet cells was set to 1.0. (TIF) [file pone.0154614.s004.tif]

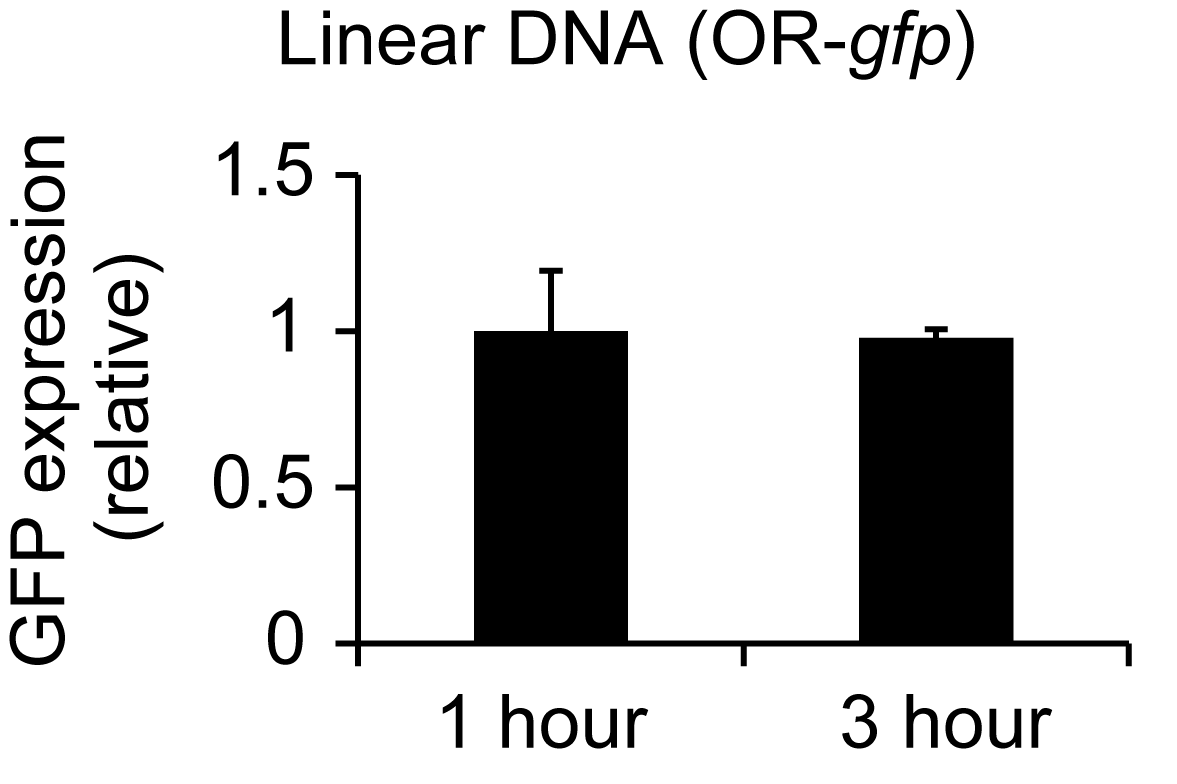

Supplement: S5 Fig — Relative GFP expression levels during the CFPS reaction were plotted. CFPS was performed at 29°C for 1 h or 3 h. Expression levels of sfGFP were normalized to the average value of the “1 h” condition. Error bars indicate standard deviation (n = 4). For template DNA, 5 nM of PCR product of pOR2OR1-sfGFP-T500 was used. (TIF) [file pone.0154614.s005.tif]

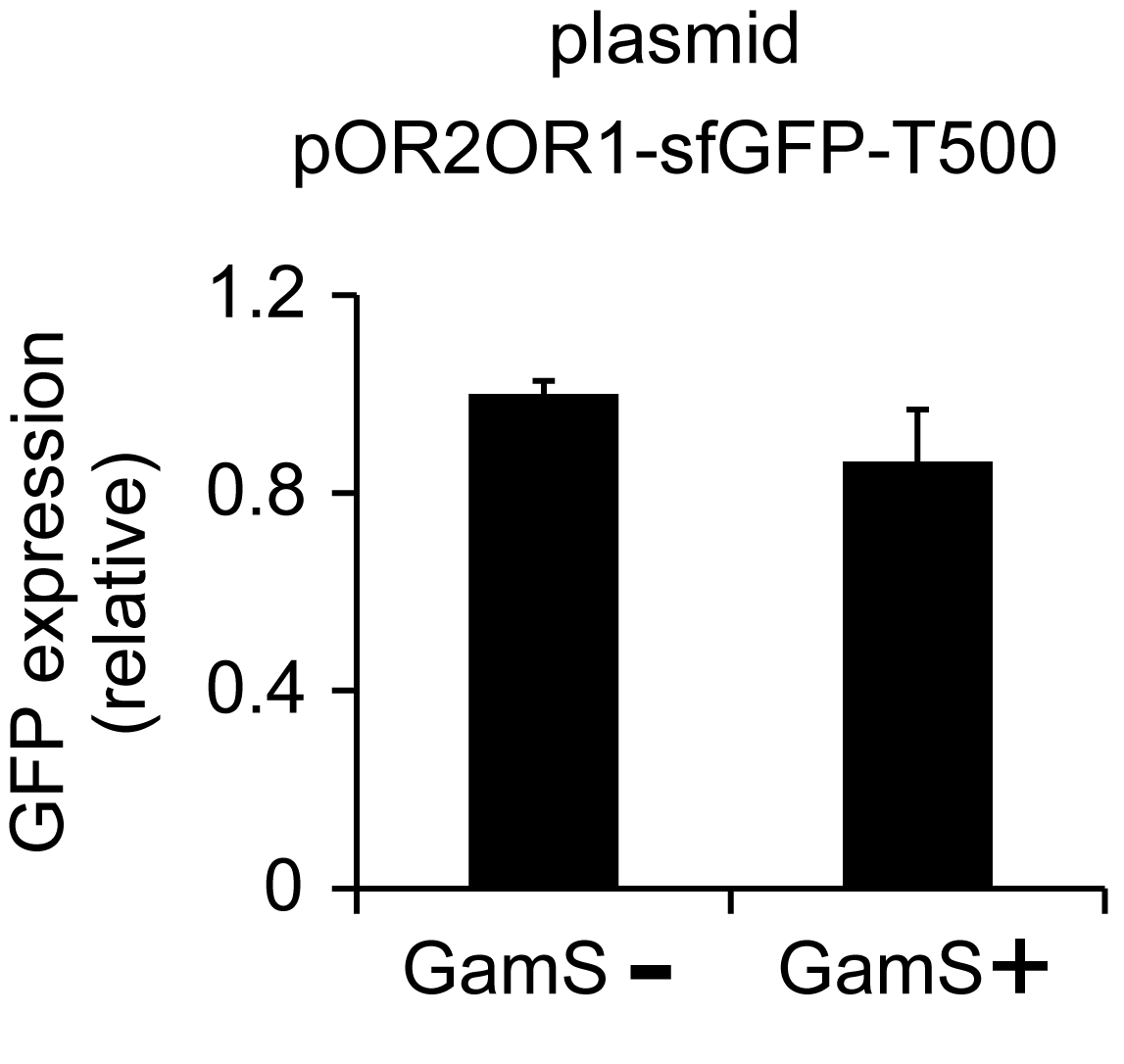

Supplement: S6 Fig — Relative sfGFP expression levels after the CFPS reaction 29°C for 14 h with or without GamS are shown. Expression levels of sfGFP were normalized to the average value of “GamS-.” Error bars indicate standard deviation (n = 4). (TIF) [file pone.0154614.s006.tif]

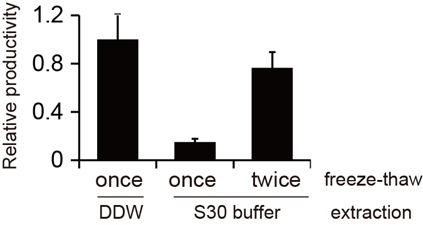

Supplement: S7 Fig — Productivity was assessed by measuring the levels of sfGFP expression after CFPS using LoFT cell extracts and 10 nM pOR2OR1-sfGFP. Error bars indicate standard deviation (n = 3). Expression of sfGFP, in the LoFT cell extract using DDW, was set as 1. (TIF) [file pone.0154614.s007.tif]
