# Supplementary material for: Biochemical Preparation of Cell Extract for Cell-Free Protein Synthesis without Physical Disruption
Source: PLoS One. 2016 Apr 29;11(4):e0154614. doi: 10.1371/journal.pone.0154614 (PMC4851396; doi:10.1371/journal.pone.0154614)
Supplement: S1 Table — (DOCX) [file pone.0154614.s008.docx]

| name | sequence |
| --- | --- |
| FtsZ-N | 5'- GGGGGGCATATGTTTGAACCAATGGAAC -3' |
| FtsZ-C | 5'- GGGCTCGAGTTAATCAGCTTGCTTACGCAG -3' |
| ORpFw1 | 5'- GGCGGTGATAATGGTTGCCTCGAGAAATAATTTTGTTTAACTTTAAGAAGGAG -3' |
| ORpRv | 5'- GTCGACTCTAGAGGATCACAGAAAAGCCCGCCTTTCGGCGGGCTTTGGTGGCAGCACCAACTCAGC -3' |
| ORpFw2 | 5'- TCGGTACCCGGGGATCTGAGCTAACACCGTGCGTGTTGACAATTTTACCTCTGGCGGTGATAATGGTTGC -3' |
| T7pUp | 5'- CCCGCGAAATTAATACGACTCAC -3′ |
| T7tDown | 5'- CAAAAACCCCTCAAGACCCGT -3′ |
